# Understanding the impacts of missense mutations on structures and functions of human cancer-related genes: A preliminary computational analysis of the COSMIC Cancer Gene Census

**DOI:** 10.1371/journal.pone.0219935

**Published:** 2019-07-19

**Authors:** Sony Malhotra, Ali F. Alsulami, Yang Heiyun, Bernardo Montano Ochoa, Harry Jubb, Simon Forbes, Tom L. Blundell

**Affiliations:** 1 Department of Biochemistry, University of Cambridge, Cambridge, United Kingdom; 2 Wellcome Genome Campus, Hinxton, Cambridgeshire, United Kingdom; University of Michigan, UNITED STATES

## Abstract

Genomics and genome screening are proving central to the study of cancer. However, a good appreciation of the protein structures coded by cancer genes is also invaluable, especially for the understanding of functions, for assessing ligandability of potential targets, and for designing new drugs. To complement the wealth of information on the genetics of cancer in COSMIC, the most comprehensive database for cancer somatic mutations available, structural information obtained experimentally has been brought together recently in COSMIC-3D. Even where structural information is available for a gene in the Cancer Gene Census, a list of genes in COSMIC with substantial evidence supporting their impacts in cancer, this information is quite often for a single domain in a larger protein or for a single protomer in a multiprotein assembly. Here, we show that over 60% of the genes included in the Cancer Gene Census are predicted to possess multiple domains. Many are also multicomponent and membrane-associated molecular assemblies, with mutations recorded in COSMIC affecting such assemblies. However, only 469 of the gene products have a structure represented in the PDB, and of these only 87 structures have 90–100% coverage over the sequence and 69 have less than 10% coverage. As a first step to bridging gaps in our knowledge in the many cases where individual protein structures and domains are lacking, we discuss our attempts of protein structure modelling using our pipeline and investigating the effects of mutations using two of our in-house methods (SDM2 and mCSM) and identifying potential driver mutations. This allows us to begin to understand the effects of mutations not only on protein stability but also on protein-protein, protein-ligand and protein-nucleic acid interactions. In addition, we consider ways to combine the structural information with the wealth of mutation data available in COSMIC. We discuss the impacts of COSMIC missense mutations on protein structure in order to identify and assess the molecular consequences of cancer-driving mutations.

## Introduction

Cancer is one of the most common diseases afflicting humanity today and the second leading cause of death globally (WHO Key Facts, Feb 2018). Cancer refers to any genetic disease that leads to an uncontrolled proliferation, causing a tumor. In 2015, there were 90 million cases worldwide and 8.8 million deaths due to cancer[1]. Its toll on the world is expected only to increase in the future.

Drug development is an expensive and time consuming process that can take decades, but the first step for most cancers is to look for a good protein target. Thanks to many breakthroughs in the field of human genome sequencing, we now have a vast amount of information that may improve our understanding of the genetics of cancer. Although we have a good description of mutations that recur in common cancers, defining the structures of the gene products, which is important for predicting the impacts of most mutations, is much more challenging and expensive. This leads to a gap in our understanding of how the sequence data relate to the structure and function of the protein.

In 2003 when the human genome project first sequenced the entire human genome, it cost an estimate of $300 million and a world spanning initiative (https://www.genome.gov/27565109/the-cost-of-sequencing-a-human-genome/). Since then many improvements and breakthroughs have been made in the field of DNA sequencing, drastically decreasing its cost and time consumption. In 2015, the cost of generating a high quality sequence of the whole human genome had fallen to below $1500, and the time required had dropped from 13 years to just 1 or 2 days[2]. These technologies have been collectively termed second generation or next generation sequencing.

The reduced cost in genome sequencing has allowed researchers to find trends in mutations in many tumors taken from patients around the world. Along with the cheaper sequencing technology has come the need for online databases to store sequence data. COSMIC[3], the Catalogue of Somatic Mutations in Cancer, is currently the most comprehensive database of mutations in cancer. Started in 2004, COSMIC provides curated information on somatic mutations. It combines large scale genome screening data from over 32,000 genomes (v86, August 2018), and manual curation of over 25,000 individual publications.

An important focus of the manual curation in COSMIC is the Cancer Gene Census (CGC) [4], a list of genes with substantial literature describing their impacts in cancer development, diseases caused, and indications of the mechanisms involved. There are currently 719 genes in the Cancer Gene Census, which are causally implicated in oncogenesis, and are divided into tier1 (574) and tier2 (145) types. For a gene to be included in the census, there has to be genetic evidence from two or more independent reports showing mutations in the gene in primary patient material, and ideally biological information supporting the oncogenic effects of the mutations. Tier1 genes have a documented activity relevant to cancer, and the mutations in the gene product promote oncogenic transformation and change the activity of the gene product, whereas for the genes in tier2 there is less evidence for their roles in cancer. The census does not include genes that experience only altered levels of expression in cancer cells, or genes that experience epigenetic changes such as methylation of CpG dinucleotides within promoter regions. These are likely the consequences rather than determinants of the oncogenesis.

### Protein structures for Cancer Gene Census analysis: COSMIC-3D

To have a better understanding of the structural and functional impacts of the cancer-related mutations, it is important to map these mutations on to the protein structure and analyse their interactions with other cellular macromolecules (such as proteins, nucleic acids, ligands etc.).

COSMIC-3D (http://cancer.sanger.ac.uk/cosmic3d/) provides a new bioinformatics platform for analyzing mutations in some of the 9300 genes in COSMIC including the 390 genes from the Cancer Gene Census onto the experimentally-derived human protein structures[5]. By mapping the mutation data onto the crystal structure of the protein, COSMIC-3D provides a helpful route to understand the structural context of the mutation in terms of its interaction with the other residues in the same protein or with other molecules when the structure of the protein is available in a bound conformation. However, as not all mutations will directly impact on interaction interfaces, further predictive tools are required. The first challenge is to use the experimental data brought together in COSMIC-3D to understand further the impacts of different missense (nonsynonymous) mutations from COSMIC.

### Identifying driver mutations

Cancer originates from genetic alteration(s) that affect cellular processes and division. Genes that are highly mutated and lead to cancer progression are known as drivers, which can be characterized as either oncogenes (activating) or tumor suppressors (inactivating)[6]. Candidate driver genes have often been identified based on mutation frequency of that gene compared to the background mutation rate, which is very challenging to estimate due to variability between cancer samples and cancers type[7].

There are three ways that are commonly used to identify background mutations: first frequency-based approaches[8] based on synonymous mutation rates; secondly, feature approaches, such as guanine and cytosine (GC) content, gene density, nucleosome occupancy, distance to telomere and centromere *etc*.[9]; and thirdly function-based methods that consider mutations in the conserved region of the protein that might have functional impacts[10], estimated on the basis of chemical and structure similarity between wildtype and mutant amino acid. The number of samples does not matter in the functional assay unlike frequency estimation methods[8].

Estimation of synonymous mutation rates is problematic where genes have very small numbers of synonymous mutations; here the rates can be estimated by mutations occurring at intron and unrelated regions assuming mutations occur there naturally, which is not always true. Driver genes are difficult to identify either by the background frequency rate or functional-based methods. This is mainly because, when there are several other genes present in the same pathway, mutation of the first gene could give a selective advantage for a tumor to progress, and therefore, other gene mutations will infrequently act as drivers[11]. Although most of the cancer-driver genes are associated with one cancer type, there are a few genes present in more than one cancer type such as TP53[12]. A new PanCancer study has identified a total of 299 driver genes using multiple bioinformatics algorithms[13].

Identification of driver mutations in the patient genomes from a set comprising all occurring mutations is a daunting task and needs functional tests that are usually time consuming and laborious. Hence, the driver mutations are usually identified on the basis of their recurrence at a particular position in all samples. The ones with highest frequency are usually identified as likely driver mutations. Where the mutation frequency is not helpful, possible drivers are often suggested on the basis of 3D proximity to each other or to other frequent mutations in that gene[14]. However, identification of driver mutations from the set of more prevalent passenger mutations remains an important step in the development of effective and targeted therapies towards different cancer types.

Here, we focus on understanding the effects of mutations in multicomponent molecular assemblies, found in the cytoplasm, nucleus, membranes and vesicles in the cell. This comprises a major challenge as further structural information is required to understand the impacts of mutations. Although ~500 of the 719 proteins in the CGC have an experimental structure in the PDB, less than a fifth of the experimental structures reported have 90–100% coverage of the full sequence. Furthermore, complete structures are available for very few of the multiprotein assemblies that are required for cellular function. We show that mutations in the CGC of COSMIC likely affect protein stability as well as protein-protein, protein-ligand and protein-nucleic acid interactions. The importance of mutations listed in COSMIC that affect such interactions has been emphasised in recent analyses[15–18]. In order to understand the structural and functional impacts of genes from CGC, we have predicted structural information where it is not experimentally available and mapped mutations not only onto the structures of individual domains, but also multidomain and multicomponent systems, using statistical and machine-learning methods to predict their impacts, often through allosteric mechanisms. We illustrate our approach with case studies not only where structures are experimentally defined and therefore provide a reliable basis for the predictive methods, but also where individual domains or subunits are defined, and full-length proteins or multicomponent systems need to be modelled. We have selected examples that include the impacts of mutations on protein-protein (Ras with Son of Sevenless homolog protein, SMAD2 homodimer), protein-ligand (BRAF-inhibitor complex) and protein-nucleic acid (androgen receptor) interactions in important cell regulatory systems. This approach adds to our understanding of cancer target function and helps in distinguishing functionally important mutations.

## Results

### Mapping sequence domains to the genes in the Cancer Gene Census

**Fig 1:**
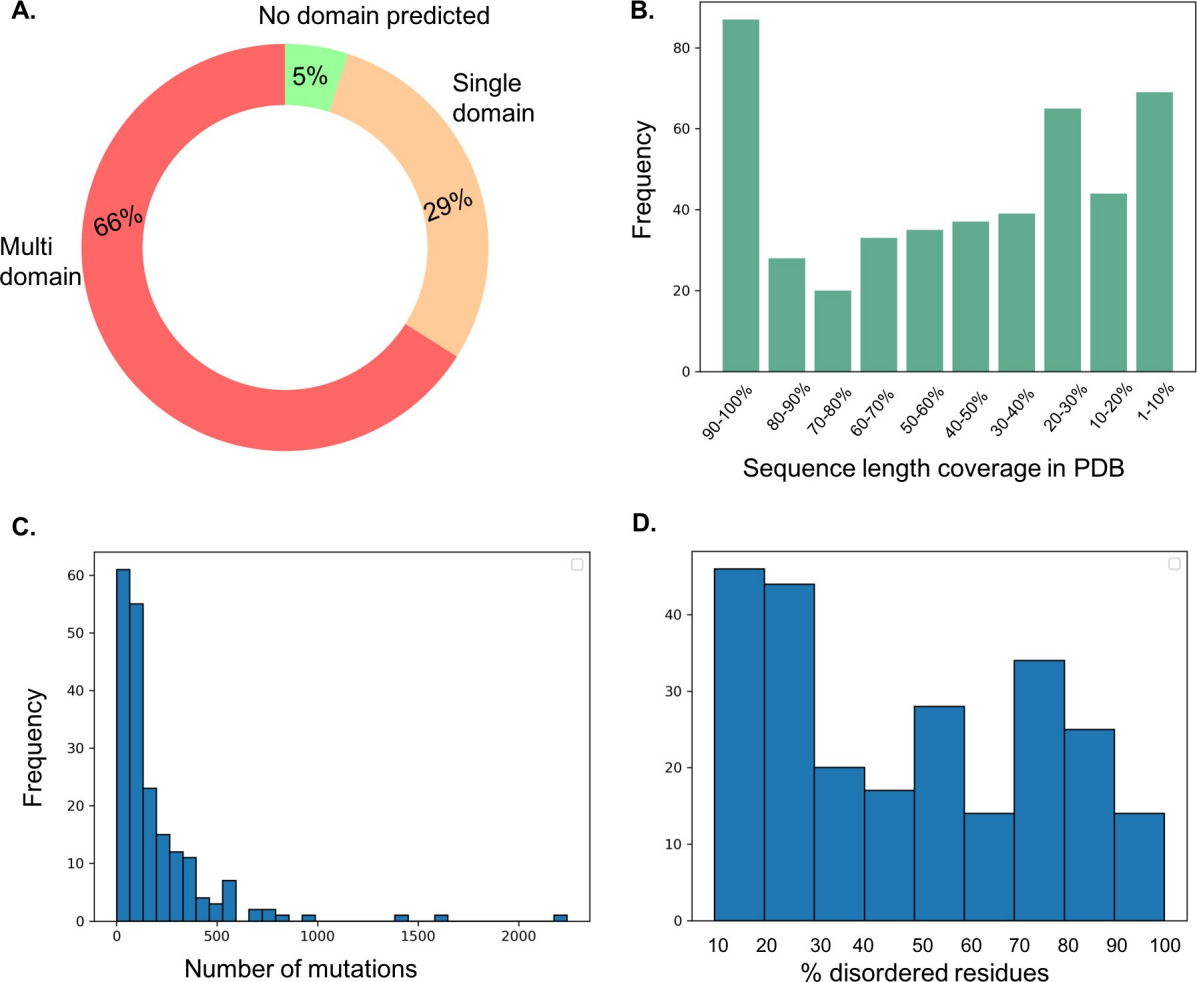
A. Mapping sequence domains to genes in the cancer gene census. 66% of the genes are predicted to have multiple domains and 29% of the genes are single domain. B. The genes in the Cancer Gene Census with their structural coverage in the protein databank. C. The distribution of mutations reported in the genes from Cancer Gene Census that do not have structure representation in the Protein DataBank. D. Histogram showing the distribution of residues in disordered regions for the protein sequences of the genes from Cancer Gene Census that do not have structure representation in the Protein DataBank.

In order to gain insights into the functions and impacts of mutations of the proteins with unknown structure, where possible we map sequences of gene products to sequence domains using an HMM[19] search against the PFam[20] database. Of the 719 genes included in the Cancer Gene Census, 205 genes are single domain and 476 genes are predicted to be multi-domain, leaving 38 genes with no PFam domain predicted using HMMER3 (Fig 1A). Furthermore, many are either homo-oligomers or contributors to multicomponent assemblies, often varying over space and time. This presents a major challenge to experimental approaches, which many of us are pursuing. However, computational analyses of structures and their interactions will likely be required for many years to come.

### Beyond COSMIC-3D

COSMIC-3D is severely limited by the availability of experimentally solved protein structures present in the PDB. Of the 719 genes in the CGC, 469 have a structure representation in the PDB, leaving 250 without PDB structures. This number of available structures is further limited by the fact that most often the structure solved for a gene product does not have a100% coverage of its sequence. In the set of 469 genes with known structures, only 87 structures have 90–100% coverage over the sequence (see Fig 1B) and 69 have less than 10% coverage. Furthermore, many of the 250 genes with no structure representation have a large number of mutations documented in the CGC (Fig 1C). We have also assessed the protein sequences of these 250 genes with no known structure information in terms of their disorder content (Fig 1D) using DISOPRED3[21], and have shown that many have a high percentage of residues in disordered regions (5% of genes have >90% residues in disordered regions). Clearly, to interpret the effects of these on protein function, one needs to build structural models. We are in the process of organizing these structural models and the predicted effects of mutations in the form of a database (Alsulami AF, P. H. M. Torres and Blundell, TL, under preparation).

### Effects of mutations on protein structure and function

Owing to their genetic instability the cancer samples are highly heterogeneous and possess many missense mutations. However, most of these mutations are likely to be neutral or passengers; only a few have deleterious effects and are driver mutations under positive selection pressure. Both oncogenes and tumor suppressor genes are involved in a dense network of interactions with other proteins, nucleic acids and small molecules. Therefore, by combining knowledge of the mutation data with information on molecular interactions, we can identify the molecular mechanisms of carcinogenesis and the likely impacts of mutations in driver genes [22,23].

Recently, there have been attempts to map the mutation data from different cancer-cell types onto protein structures and hence identify clusters of mutations[14,24]. This helps in identifying the new targets, types of interactions disrupted upon mutations and the potential functional effects. However, experiments in structural biology and mutagenesis studies comparing the free energy differences between wildtype and mutant proteins are costly and time consuming. Nevertheless, databases such as ProTherm[25] provide a resource for experimental thermodynamic data on mutant proteins, allowing for larger scale studies of mutation impacts.

This has led to the development of many computational algorithms to study missense mutations and their impacts on protein stability and function. There are several different approaches that have been used to study the impacts of mutations. Most sequence-based approaches consider the local conservation patterns in homologues to predict how damaging mutations at a certain residue would be. SIFT[26] and PolyPhen[27] are very popular sequence-based methods. Structure-based approaches, which make use of the protein 3D structure (either experimentally derived or modelled), typically fall into the categories of potential energy functions or machine-learning methods. The physics-based methods of predicting the effects of mutations rely heavily on the position of side chains in order to define interactions and clashes; therefore, they require accurate positioning of the atoms including bound waters. On the other hand methods that are based on either statistical potentials, such as BeATMuSiC[28], or rely on structural profiles[29] usually require only an approximation of the protein structure. STRUM[30] shows that the predictions of effects of mutations rely more on the accurate prediction of the global fold and have only a marginal dependence on the accuracy of protein structures.

Some early methods such as SDM[31,32] use environment-specific substitution tables, while others such as PoPMuSiC[33,34] use potential energy functions to calculate the change in free energy. More recently, some structure-based approaches, such as mCSM, have used machine-learning methods. These can come in different flavours such as mCSM-PPI[35] (protein-protein interactions), mCSM-lig[36,37] (ligand binding) and mCSM-NA[38] (nucleic acid binding) or neural networks (PoPMuSiC-2) to predict the impact of mutations. There are various ways of feeding structural data into a machine-learning algorithm; one approach has been to turn the structure into a graph-based signature, which is the principle behind mCSM.

There are also molecular dynamics-based methods that can predict the effects of single point mutations at protein-protein interfaces[39]. Kellogg *et al*. have investigated the performance and accuracy of different protocols to predict effects of mutations by extensively searching through alternative conformations[40]. Such analyses of the wealth of mutation data in tumors, using these various mutation-analysis softwares, can be used to evaluate their ability to predict carcinogenic mutations.

### Preliminary studies of Cancer Gene Census proteins

Hallmark genes in the CGC are classified following the approach of Hanahan and Weinberg [41], exemplifying the following biological capabilities: proliferative signalling, suppression growth, escaping immune response to cancer, invasion and metastasis, tumor promoter inflammation, and cell replicative immortality. Hallmark genes are marked in the CGC and have manually curated information available on protein function.

In order to illustrate the challenge of understanding the impacts of the mutations we have extended the modeling to examples from the Hallmark dataset in the Cancer Gene census as these have a huge amount of manually curated information on the functional effects of mutations that will help in understanding the structural changes upon mutations. We have chosen five examples of proteins that have features characteristic of the Cancer Gene Census. These include structures that are experimentally defined and therefore provide a more certain basis for assessing the impacts of the modelling software; they also include modelled structures where predictions of protomer structures are generally reliable, but where multicomponent assemblies are more challenging. They include homo-oligomeric structures, hetero-oligomers/ multiprotein assemblies with protein-protein interactions, nucleic acid-binding proteins, protein-ligand interactions and membrane proteins.

## Multiprotein assemblies

### Hetero-complex of Ras protein with Son of Sevenless protein homolog 1

Ras is a signalling molecule that acts as a switch by shuttling between the active (GTP bound) and inactive (GDP bound) form. Ras forms a complex with Ras-specific nucleotide exchange factor, Son of Sevenless (SOS), which helps in activating receptors that signal through tyrosine kinases. It is known that the unregulated activation of Ras is a hallmark of many cancers[42].

**Fig 2:**
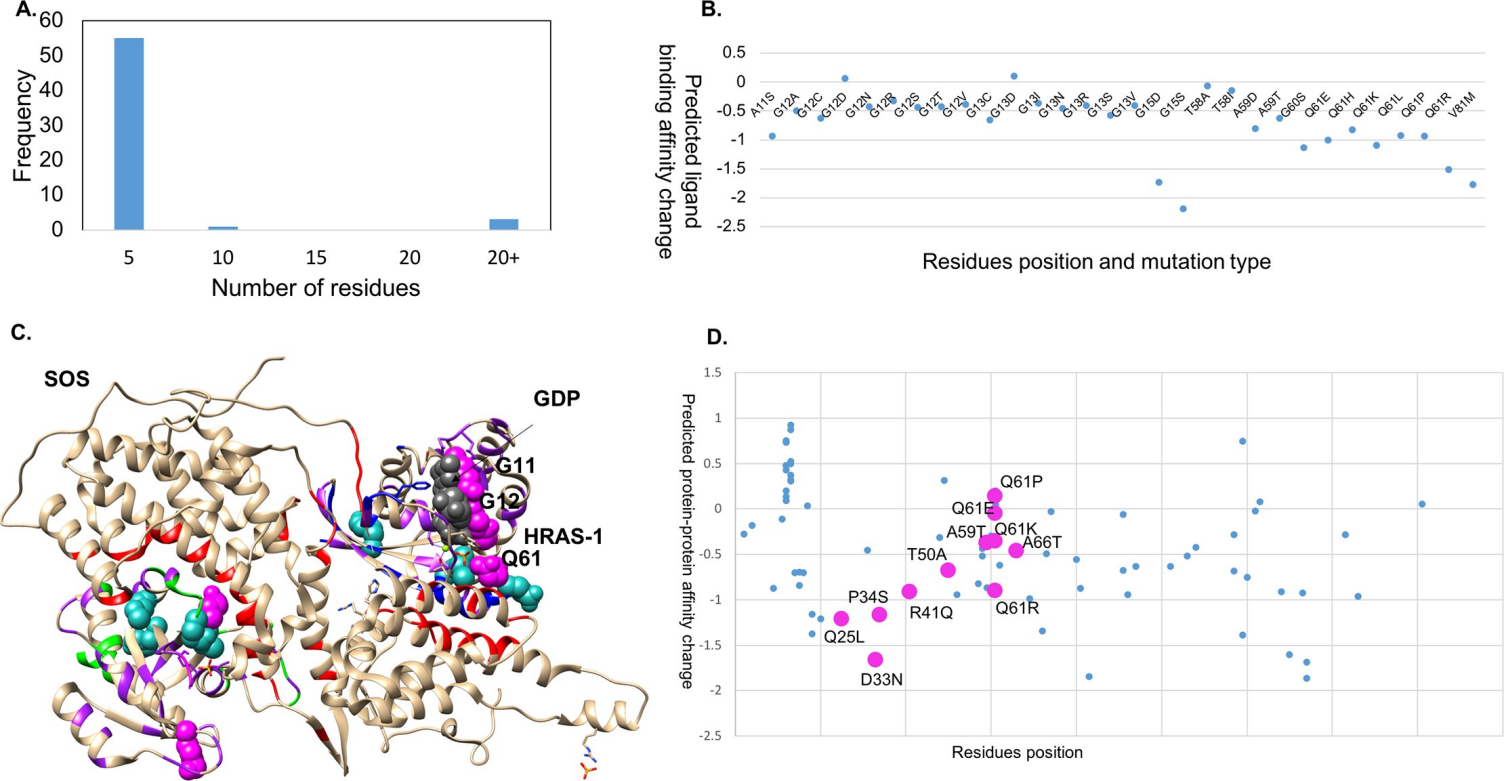
Mutations in HRAS-1. A. Frequency distribution in cancer samples in COSMIC. C. Mutations mapped on to the hetero-complex of HRAS-1 with Son of Sevenless. The driver mutations are colored in magenta (recurrence > = 10) and sea green (recurrence > = 3). B. mCSM-lig values for the mutated sites. D. mCSM-protein-protein values for the interface residues. The residues present within the 5Å of the ligand binding site (GDP), are highlighted as pink circles.

Here, we have mapped the mutations known for HRAS-1 from COSMIC onto the hetero complex of SOS with HRAS-GDP (PDBID: 1XD2[42]). For HRAS-1, 59 residue positions are documented to have mutations in at least one cancer sample (Fig 2A). We have mapped the mutations known for HRAS-1 from COSMIC onto the ternary complex of SOS with HRAS-GDP (PDBID: 1XD2). The most frequently mutated residues are G12, G13 and Q61. Gao *et al*. [24] have recently identified mutational 3D clusters, which assist in identifying the possible driver mutations in cancer targets. Q61 was observed to be part of one such cluster, which has other residues with low mutation frequency (colored in purple) and these spatially close residues are within 5Å of the ligand binding site (GDP), highlighting their functional role (Fig 2B and 2C).

We used mCSM-PPI[35], trained on effects of mutations on protein-protein interfaces, to predict the effects for the interface residues (between HRAS-1 and SOS), which are reported to have mutations (eight residues including Q61, shown as magenta in Fig 2D) in COSMIC. The majority of the interface residues were observed to have destabilizing effects for the interface. Q61, present within 5Å of the ligand GDP, is the most recurrent mutation, with a count of 659 and was observed to be mutated to seven other residues, which are all predicted to reduce ligand binding affinity using mCSM-lig (Fig 2B). Three of these (Q61R, Q61K and Q61E) are predicted to have destabilizing effects on protein-protein interactions (Fig 2D). Hence, on the one hand the driver mutations have impacts on both protein-protein and protein-ligand interactions, but on the other, because mutating functionally important residues comes with a fitness cost, the changes are to amino acids with ddG values close to zero.

### Homo-dimers SMAD2

Smad2 is a receptor-regulated Smad (R-Smad), a functional class involved in ligand specific, TGF-β-cell-signaling pathways and implicated to function as tumor suppressors[43]. The pathways involving TGF-β are known to regulate cell growth, proliferation, apoptosis, differentiation and developmental pathways[44] and are initiated by cytokine binding to the TGF-β transmembrane receptors, kinase activation, recruitment of a specific R-Smad and phosphorylation of the SSXS motif (pSer motif) at the C-terminal, formation of a hetero-oligomer of R-Smad and Smad4 (ubiquitous, comediator Smad)[43,45]. The hetero-oligomer regulates the expression of genes in the nucleus in response to the specific ligand. Mutations in this pathways are known to cause human cancer and developmental disorders[4,46].

Although the unphosphorylated Smad2 is a monomer, phosphorylated Smad2 is known to form a homotrimer both *in vitro* and *in vivo*[43]. The phosphorylated C-terminus of one protomer contacts the L3/B8 loop-strand pocket of the adjacent protomer, using the two pSer residues as anchors. The residues that mediate interactions in the homotrimer are observed to be conserved in Smad4 and also there have been suggestions that the same surface of Smad2 is used to form a hetero-complex with Smad4[43,45].

**Fig 3:**
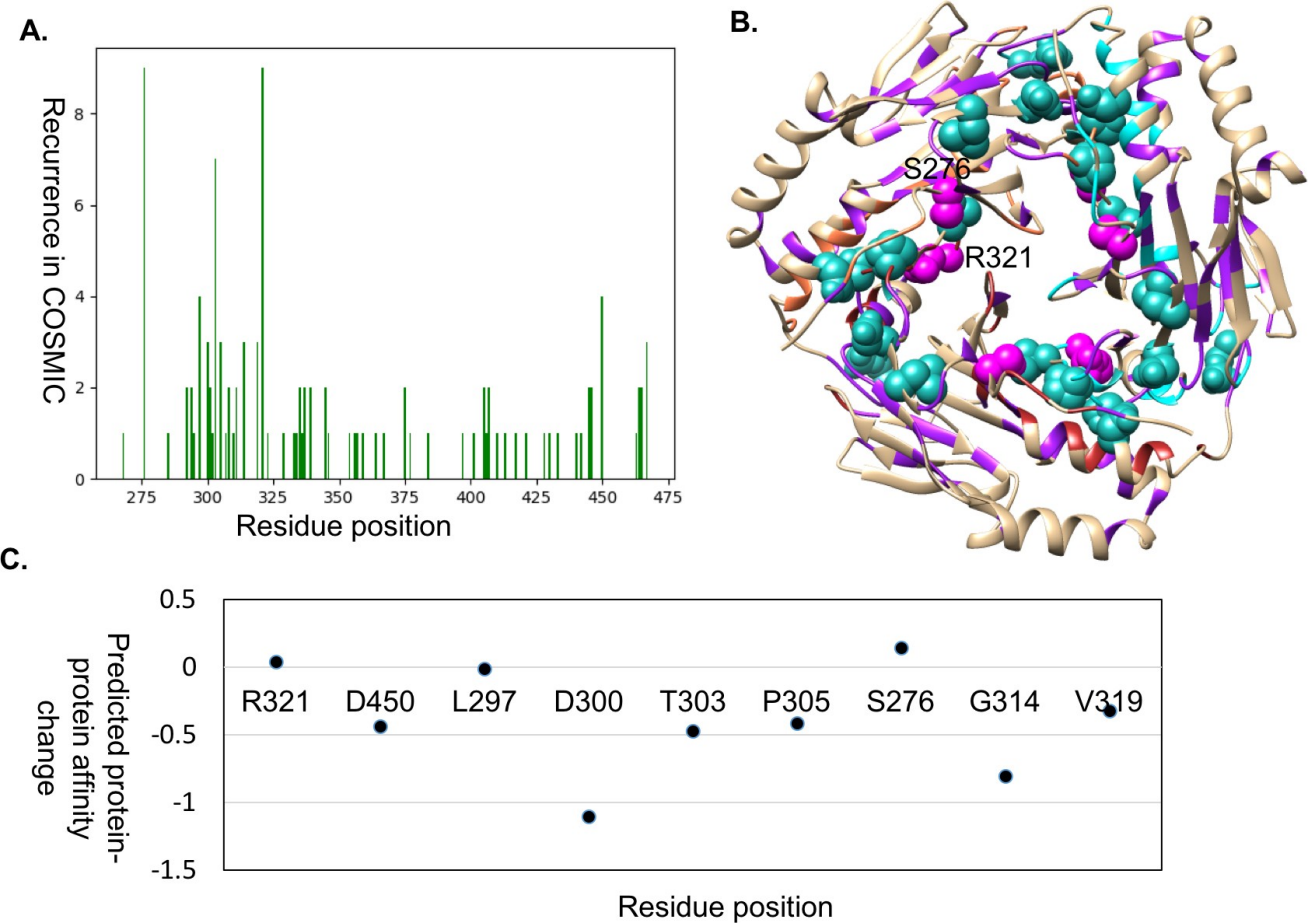
Mutations in Smad2. A. Frequency distribution in cancer samples in COSMIC. B. Mutations mapped on the homotrimer of Smad2. The residues that are documented to have mutations are shown in purple ribbon, and the driver sites are marked in sea green (at least three times) and magenta (more than three times) spheres. C. mCSM-protein-protein values for the most frequently mutated interface residues.

We used the structure of the central domain (MH2 domain) (PDBID: 1KHX, 1.8 Å) to map the mutations from COSMIC. There are nine mutations that occur at least three times (Fig 3A), and are seen at the protein-protein interface of the heterotrimer (shown in magenta and green spheres in Fig 3B), other than S276, which is at the core of the MH2 domain of Smad2 and is responsible for the structural stability of each protomer. Most mutations at the residue positions were predicted to have a destabilizing effect at the protein-protein interface (Fig 3C) using mCSM-PPI.

Hence, the mapping of the cancer related mutations onto the surface of the MH2 domain of Smad2 implies that these mutations alter the formation of homo/hetero complex formation and hence might affect the tumor suppressor roles of Smad proteins. A similar spectrum of mutations is discussed for Smad4 and Smad3[46,47].

### Small-molecule ligand-binding proteins

#### BRAF-MAP2K1

RAF kinases are Ser/Thr kinases known to have three isoforms in human: ARAF, BRAF and CRAF. They are involved in the MAPK (Mitogen-Activated Protein Kinase) pathway, which plays role in cell growth, ageing and differentiation. RAF kinases are activated by the GTP-bound RAS molecules, which in turn phosphorylate MAP kinases resulting in downstream cell signaling towards cell cycle progression and transformation. RAF molecules are known to have mutations that are implicated in human cancers due to their constitutive activation.

The most frequently mutated residue in BRAF (V600) is located in the kinase domain close to the sites of phosphorylation (T599 and S602[48]), which are responsible for the downstream signaling. The mutation V600E is reported to mimic the effect of phosphorylation as it has higher kinase activity than the wild type[49].

**Fig 4:**
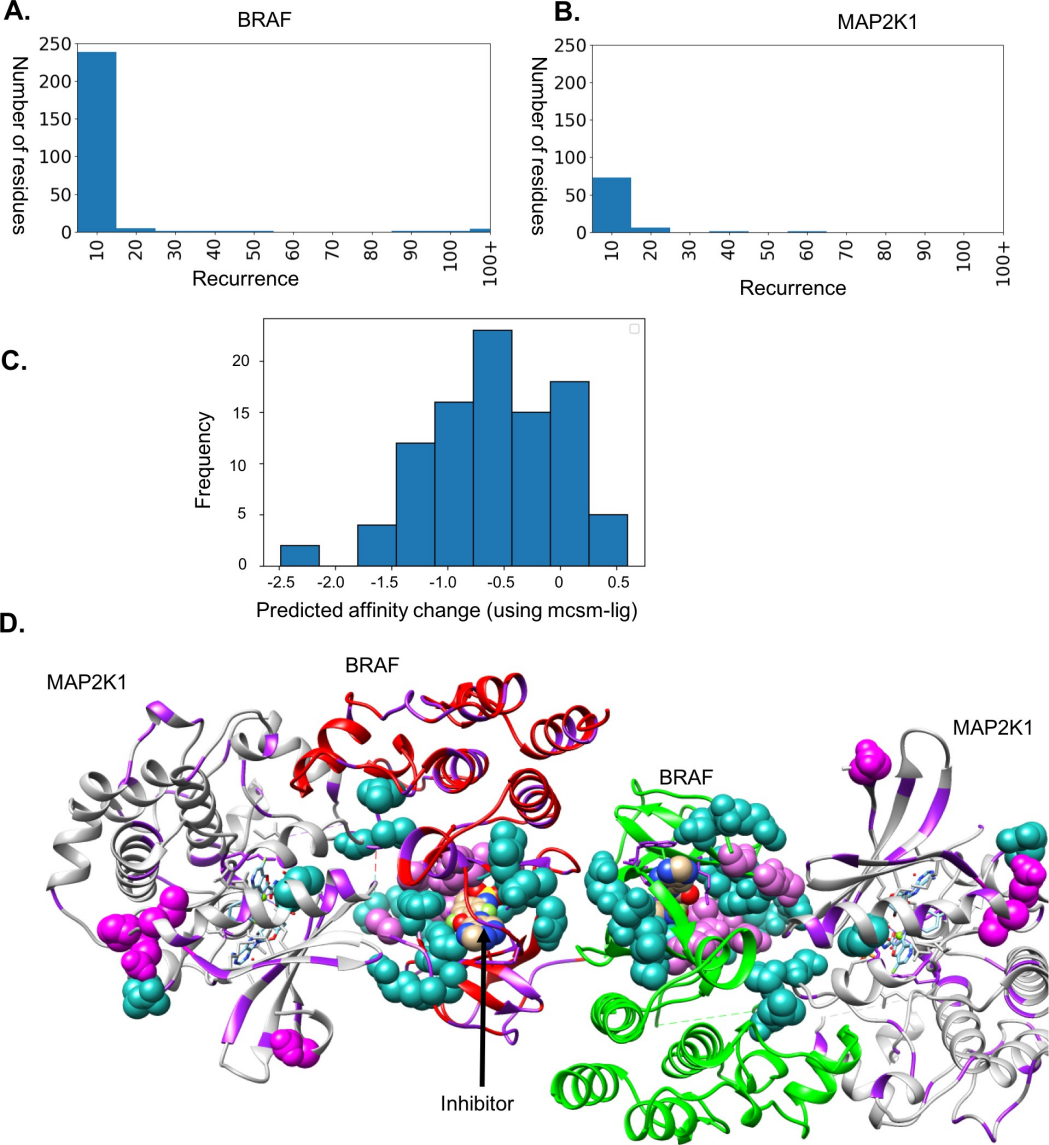
Mutations in BRAF. A. Frequency distribution in cancer samples in COSMIC for BRAF. B. Frequency distribution in cancer samples in COSMIC for MAP2K1. C. mCSM-lig values for the driver residues and protein-ligand interface residues. D. Mutations mapped on the complex of BRAF and MAP2K1. The residues that are documented to have mutations are shown in purple ribbon, and the driver sites are marked in sea green (at least three times) and magenta (more than three times) spheres.

The Cancer Gene Census in COSMIC has data from 50,176 samples and 50,742 missense mutations for BRAF (Fig 4A and 4B). We used the structure with PDB IDs: 4MBJ (protein-ligand, structure of BRAF kinase domain with an inhibitor) and 4MNE (protein-protein complex, structure of BRAF kinase domain with MAP2K1) to map the mutations to understand the functional impacts of the mutations and envisage the roles of driver mutations at protein-protein and protein-ligand interactions.

We studied the effects of mutations on the inhibitor binding on the BRAF kinase domain as the recurrent mutations were observed to be present near the inhibitor-binding site (within 5Å of the inhibitor). The majority of these were predicted to decrease the inhibitor-binding affinity and hence might contribute towards resistance (Fig 4C and 4D). The 42 residue positions are reported to have a recurrence rate of at least three (sea green Fig 4D) and 18 residue positions have a recurrence of at least ten (magenta in Fig 4D).

We identified interface residues from the complex structures of BRAF, as the residues in the biological assembly of 4MNE having a Cβ-Cβ distance of 7Å or less of the homodimer interface of BRAF and the BRAF interface with MAP2K1. There are 23 interface residues at the homodimer interface and 31 residues are at the interface with MAP2K1. Of these interfacial residues, nine residues have mutations documented in the cancer gene census (Table 1). We predicted the effects of these mutations using mCSM-PPI and most of them were predicted to be destabilizing.

**Table 1:** Prediction of effects of mutations at the protein-protein interface using mCSM protein-protein in BRAF.

BRAF-BRAF interface			BRAF-MAP2K1 interface		
Residue position	Recurrence	Average ddG (mCSM-PPI)	Residue position	Recurrence	Average ddG (mCSM-PPI)
L588	3	-0.274	S614	4	0.224
L515	1	-0.628	S616	7	-0.119
D586	9	-0.427	I617	1	-0.211
R509	1	-2.634	L618	8	-0.871
			H539	1	-1.253

The oncogenic mutations in BRAF are known to act through complicated and diverse mechanisms, for example the mutants possessing the most recurring V600E alteration increase the kinase activity of BRAF, whereas other less common mutations decrease the kinase activity but still promote the downstream phosphorylation and signalling using a CRAF-dependent pathway[50,51].

### DNA-binding proteins

#### Androgen receptor

**Fig 5:**
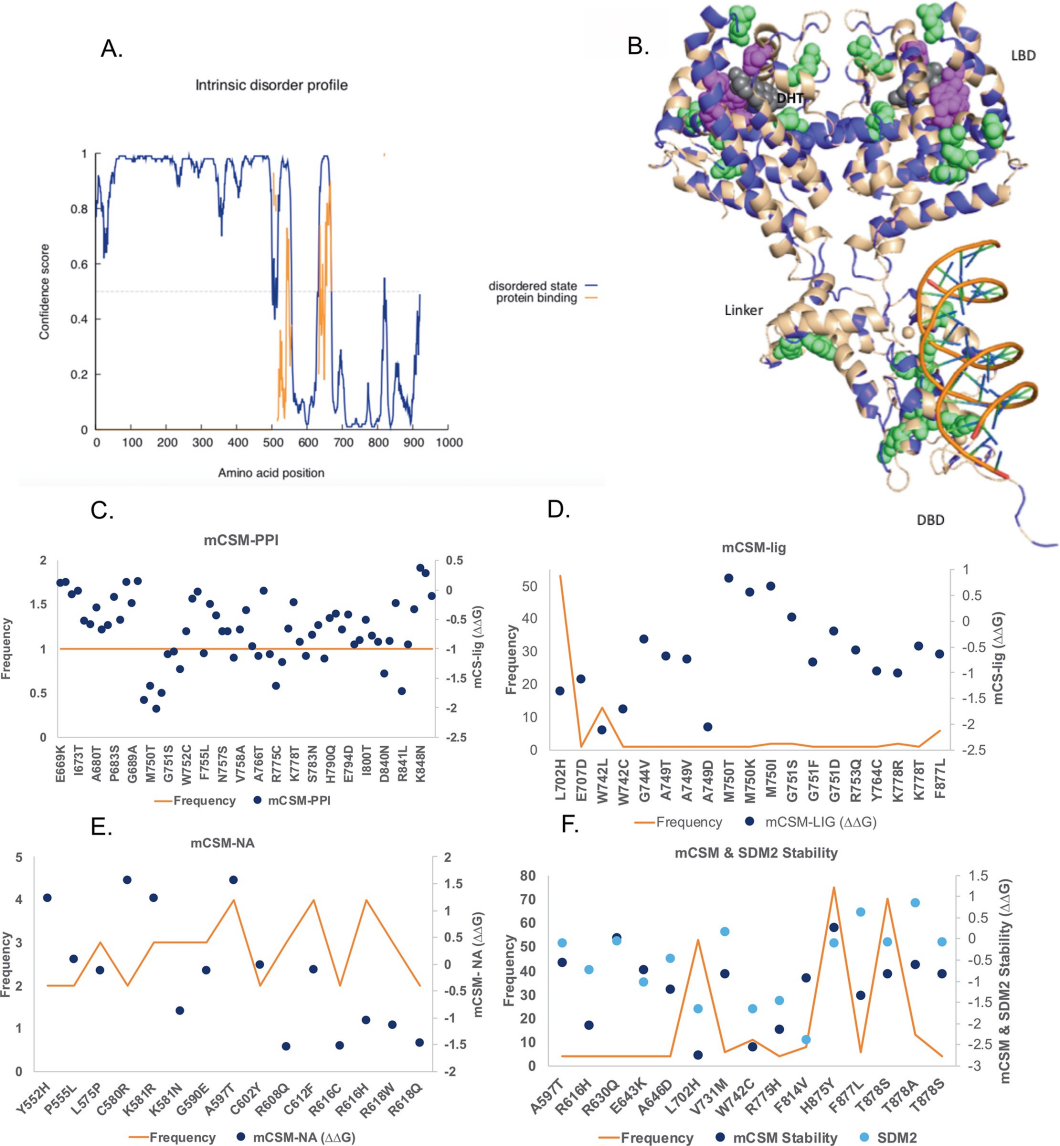
Mutations in androgen receptor. A. The intrinsically disordered region of the androgen receptor (predicted using PSIPRED). B. Missense mutations (shown in purple) from COSMIC mapped on to the modeled structure of androgen receptor homodimer structure. Driver mutation sites (recurrence > = 13), indicated by magenta spheres, are located mainly around the ligand-binding domain and the residues with a mutation frequency between 4 and 6 are shown in light green. C. The changes in protein-protein binding affinity predicted using mCSM-PPI of the residues present in the homodimer interface (from both chains). D. Changes in the ligand binding affinity predicted using mCSM-lig for the residues present within the 7Å of DHT ligand and the most recurring mutations are highlighted in magenta. E. Changes in the protein-DNA binding affinity predicted using mCSM-NA for mutations that occur within 7Å of the DNA and the most frequent mutations highlighted in light green. F. mCSM, SDM2 stability predictions of mutation reported more than 4 times in COSMIC.

The androgen receptor (AR), a member of the steroid hormone nuclear receptor family, plays an important role in sexual differentiation and also has many important biological roles such as the development of the cardiovascular and immune systems. AR signaling is also reported to have a role in the development of tumors, and is an important target for prostate cancer[52]. AR has two main domains: ligand-binding domain (LBD, residue range: 668–918)[53], which binds 5-α dihydrotestosterone (DHT) and activates downstream signaling including phosphorylation of the second messenger signaling cascade and DNA-binding domain (DBD residue range: 538–629) [54], which regulates the target gene expression[55]. The N-terminal region of AR (~ 500 amino acids) is intrinsically disordered and has no defined 3D structure (Fig 5A). The structures of both these domains have been solved independently but a linker region (residue range: 630–667) does not have a structure and was modeled using (PDB IDs; 5CJ6 and 2AM9) as template and subsequently we used Modeller to link the DNA binding domain and ligand binding domain together.

There are four types of mutations observed in the AR receptor: missense substitution, insertion or deletion, partial gene deletion, and intronic mutation. We focused on the missense mutations[56], for which there are 482 unique mutations reported in COSMIC for AR, 221 unique mutations from 789 samples were mapped to the AR LBD, DNA-binding domain and linker between the two (Fig 5B, shown as purple). Most of the frequently observed mutations cluster around the ligand-binding pocket (DHT (5-aplha-dihydrotestosterone, shown in grey) binding site (Fig 5B). We predicted the effects of these mutations on the protein stability using mCSM and SDM2 (Fig 5C). The majority of the frequently observed mutations (magenta circles) were predicted to destabilise the protein.

As the LBD of AR is homodimeric, we measured the effects of the mutations on the protein-protein interactions using mCSM-PPI (Fig 5C). All mutations in the ligand-binding domain within 7Å of any atom of an interface residues between the two chains were predicted to be destabilizing the protein-protein interactions. Using mCSM-lig we estimated the impacts of mutations within 7 Å of the ligand on the ligand (DHT)-binding: they were all predicted to have detabilising effects on ligand binding (Fig 5D). These destabilising mutations cluster mainly around the ligand-binding site and are believed to cause perturbation by increasing the mobility of an adjacent helix [57]. Experimental evidence showed that mutation of F877L, T878, H875Y decrease the sensitivity of AR toward non-steroidal antagonists such as hydroxyflutamide, bicalutamide, and enzalutamide converting it into full agonist[57].

We also estimated the impacts of mutations on DNA-binding using the mCSM-NA software (Fig 5E) and all mutations (with a frequency between 4 and 6 and within 7 Å of DNA) were observed to highly reduce the DNA-binding affinity.

### Membrane proteins

#### Transforming growth factor beta receptor II (TGF-R2)

The TGF-R2 protein belongs to the TGF-β cytokine superfamily and regulates multiple cellular activities, playing a key role in development, tissue homeostasis, and immune modulation[58]. TGF-R2 consists of an extracellular domain (residue range: 25–130), a single transmembrane domain (residue range: 157–188) and a kinase domain (residue range: 190–541) in the intracellular region[59,60]. TGF-R2 forms a homo/heterodimer upon binding the ligand, which further triggers the TGF-β signaling pathway.

**Fig 6:**
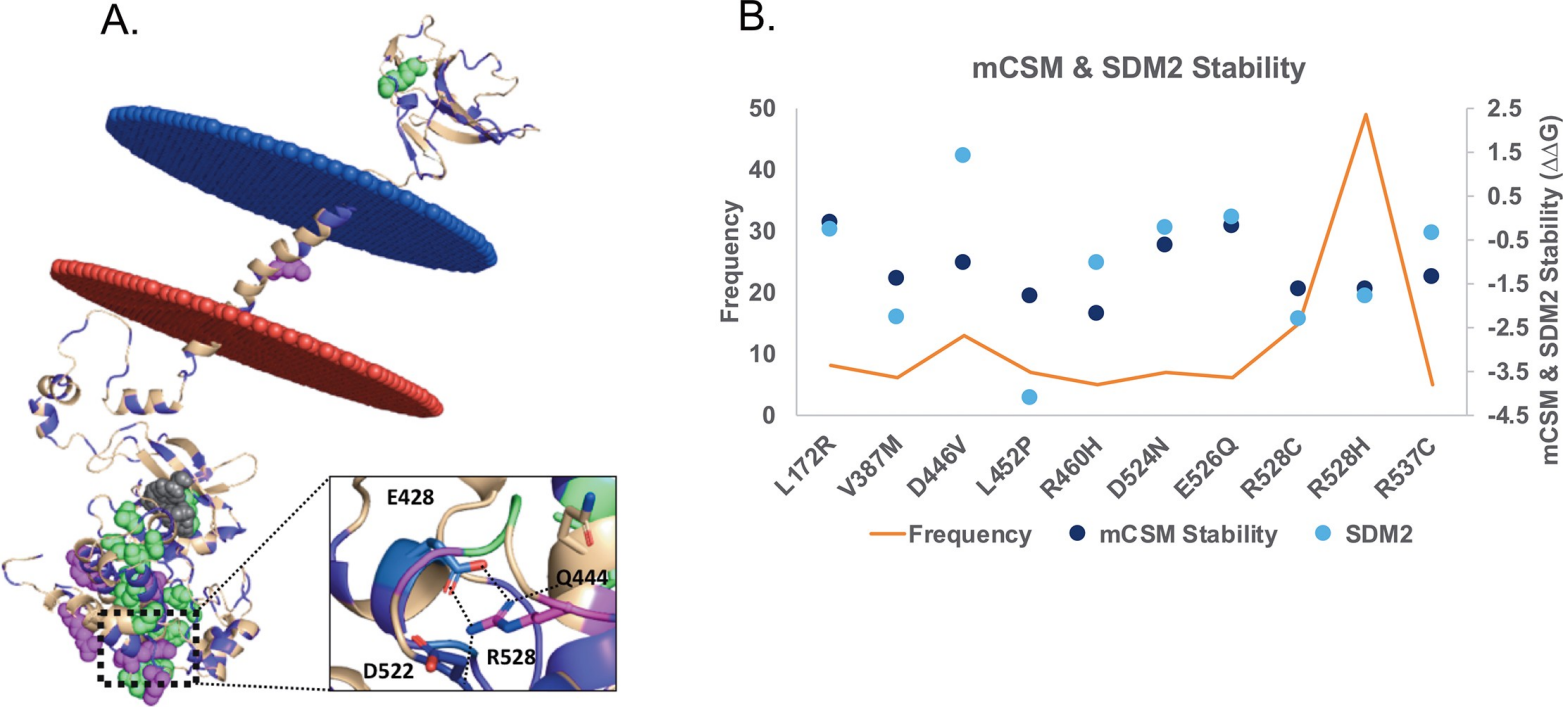
Mutations in TGF. A. Missense mutations (in purple) from COSMIC were mapped on to the modeled structure of *TGF-R2*. Residues with a mutation frequency between 4 and 6 are shown in light green spheres, and the residues with mutation frequency more than 8 are shown in magenta. The interactions of the most frequently mutated residue R528 with its surrounding residues are shown. B. The changes in the energy (ddG) corresponding to the stability of the protein structure predicted using mCSM, SDM2 for all missense mutations and the most frequent mutations are shown in magenta.

We modeled the structure the transmembrane domain and the missing regions between the kinase domain and the transmembrane residue range: 191–239, using (PDB IDs: 1H4I, 3I44, and 1H4J) as template. We then mapped the mutation data on to the modeled structure (Fig 6A). The potential driver mutations with frequencies more than eight, shown as magenta circles in Fig 6B and 6C, were predicted to have a destabilizing effect on the protein stability using mCSM (Fig 6B) and SDM2 (Fig 6C). The most frequently mutated residue R528 (67 times, to residues Gly, His, Leu and Phe) is a key residue for maintaining the stability of the kinase domain (highly buried and forms salt bridge with E428, Fig 6A). Mutating R528 to other residue types will affect the protein stability and is also predicted as highly destabilizing with mCSM and SDM2 (marked with oval in Fig 6B). Experimental evidence has shown that mutating R528 leads to conformational changes and hence alters the kinase function[61].

#### Transmembrane Pump: ATP1A1 a sodium/potassium ATPase pump

Na^+^/K^+^ ATPase, a sodium-potassium pump, expressed in all animal cells, belongs to the class IIC of P-type ATPases that utilize ATP[62]. Na^+^/K^+^ ATPase assists in maintaining the pH as well as a low sodium ion intracellular concentration and a high potassium extracellular concentration. Na^+^/K^+^ ATPase, one of the most important active transporter, works towards maintaining a resting membrane potential and signal transduction[63].

**Fig 7:**
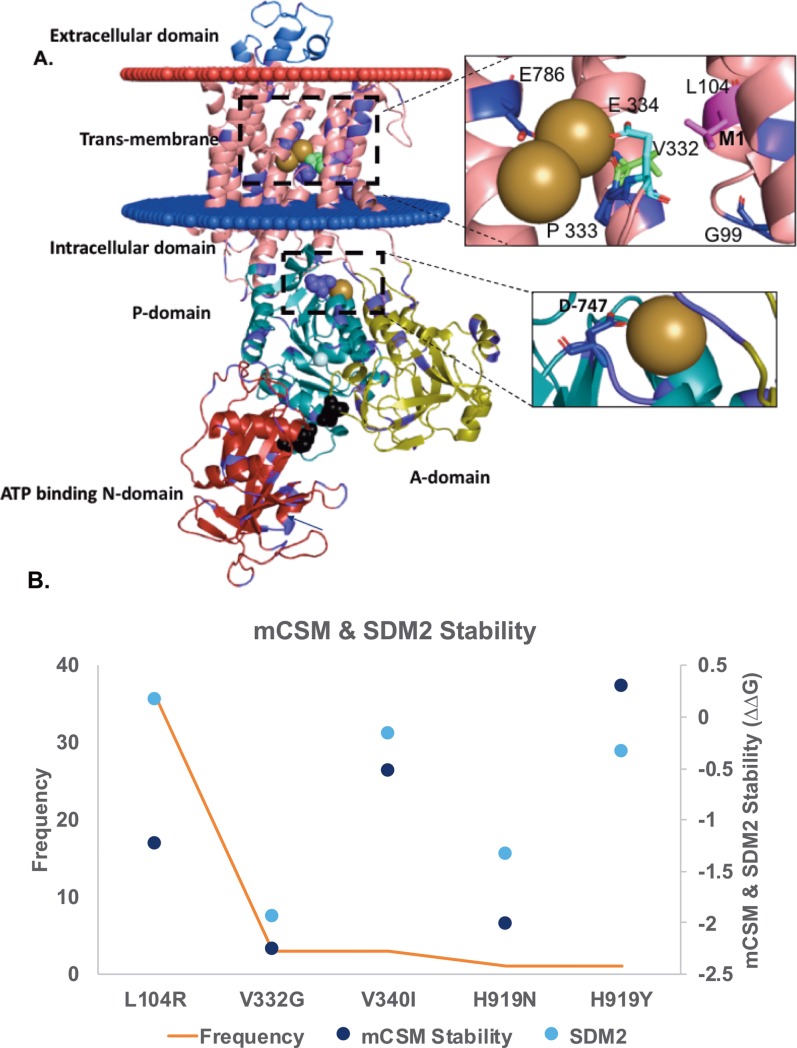
Mutations in sodium potassium pump. A. The modeled structure of sodium potassium pump ATP1A1; each domain labelled in the figure is represented in a different colour. Mutation data from the COSMIC database are mapped on to the structure in purple, the driver mutation, L104, is shown in magenta, the driver sites (recurrence > = 3) are marked in sea green. The potassium ions are shown in gold spheres and the magnesium ions in white spheres. Mutated residues within 5Å of potassium ions are shown in stick. The salt bridge connecting the N-domain to the A-domain is shown in black spheres. B. The changes in the energy (ddG) corresponding to the stability of the protein structure predicted using mCSM and SDM2.

We modeled ATP1A1 (Fig 7A) using the sodium-potassium pump (PDB ID: 2ZXE[64]) structure at 2.4AÅ resolution as a template (89% percent identity and coverage of 96%). There are three alpha-domains present in the intracellular region of ATP1A1 model: A-domain, N-domain, and P-domain. The alpha-N domain and the alpha-A domain are stabilized by a salt bridge interaction[64] between E223 and R551 (Fig 7A, shown in black spheres). ATP binding occurs close to this salt bridge and mutation of E223 and R551 will eliminate the ATP binding. Pharmacologically Na^+^/K^+^ ATPase can be inhibited by digoxin, which is used to treat heart failure. Na^+^/K^+^ ATPase has been suggested as a potential chemotherapy target for cancer[65].

150 unique mutations in ATP1A1 have been observed in 210 samples recorded in the COSMIC database. Four mutations (V332G, P333L, E786V and D747N, marked as pink circles in Fig 7A), are present within 5 AÅ of the three potassium ions (shown as gold spheres, Fig 7A). All missense mutations from COSMIC were mapped onto the Na/K model (Fig 7A). The most frequent mutation is present in the transmembrane region, L104R (M1 helix, shown in magenta, Fig 7A) has been reported 49 times and is also predicted as highly destabilizing by mCSM (ddG = -1.72) and SDM (ddG = -2.73). L292 in M3 and G99 in M1 are not observed to be frequently mutated as they function as central residues for the movement of M1 to open the gate for ions to enter into the cationic pocket, whereas E334 in M4 is part of the gate that binds to a potassium ion in the occluded stage[66] (Fig 7A). Since the movements of transmembrane domains are essential for ions to be transported in and out of the cells, mutations around the cationic pocket or in the trans-membrane region, which has to move to allow ions to be transported, will disrupt the function of the Na/K transporter. There are multiple studies on L104R mutation indicating that R104 creates a positive charge causing structure alteration around cationic pocket, as a result of which the potassium binding pocket is disrupted and cell depolarization observed[67,68]. Fig 7B, highlights that most frequently mutated residues are potential drivers predicted to have a destabilizing effect on the protein stability using mCSM and SDM (Fig 7C).

## Discussion and future perspectives

Identifying driver mutations in cancer targets is essential to guide new therapeutics. However, mutations can act at a distance from ligand binding sites not only in well-defined allosteric sites in the same subunit by changing stability but also through disturbing protein interactions with another protein, nucleic acid, metal ion or ligand. Methods to define drivers include determining their impacts on physico-chemical properties as well as understanding the roles of the mutated side chains in protein 3D structure, e.g. solvent accessibility, hydrogen bonding, and surface accessibility. There are several computational approaches to prediction of the impact of mutations on protein stability, for example SDM[31,32], and mCSM[35,36,38]. However, the lack of defined 3D structures in complexes (heterodimer, homodimer, DNA, RNA) makes it difficult to predict the impact of mutations on protein function. Usually mutations in a conserved region are recognized as drivers, whereas mutations in a non-conserved region are classified as passengers. A major challenge is to identify mutations that are outside the conserved region but lead to cancer progression. Destabilizing effects of the glioblastoma missense mutations have been observed in the protein-protein and protein-ligand interfaces[69–71]. With respect to the systems studied here most mutations appear in the interface, binding site, and between domains in ATP1A1, SMAD2, and BRAF-MAP2K1, but others can allosterically affect these interactions. It is essential to model full multicomponent complexes (heterodimer, homodimer, DNA, RNA) in order to explain the impacts on the interface, DNA, and RNA binding. These are particularly important for predictive algorithms that depend on structure such as those encoded in software such as mCSM and SDM.

Here, we have analysed the effects of the most recurrent mutations on protein-protein (hetero-Ras protein with Son of Sevenless protein and homo-SMAD2 homodimer), protein-DNA (androgen receptor with its target DNA) and protein-ligand (BRAF kinase with an inhibitor) interfaces. In the protein-ligand cases, many of the most recurrent mutations were clustered around the ligand-binding site and were predicted to decrease the inhibitor-binding affinity. Similarly, all mutations with high frequency and within the 7 Å of DNA were observed to highly reduce the DNA-binding activity. In the protein-protein complex of BRAF-kinase dimer with MAP2K1, both homo and hetero interfaces are tightly packed and comprise of 23 and 31 interface residues respectively. The mutations in these interface residues were predicted to be destabilizing and hence affect cell signalling and function.

3D hotspot clustering is one of the methods used to study driver mutations. Recently 3D structural information has been used to identify driver mutations in cancer and other diseases. Using the Fragment-Hotspot program[72] to identify druggable sites in conjunction with Hotspt3D[73], HotMAPS[74], and Mutation3D[67], which use 3D structure to identify mutation clusters in cancer should give valuable information on identifying driver mutations. The Pan-Cancer analysis has shown that structure-based approaches are more reliable but less sensitive than sequence approaches in identifying driver mutations than other methods for the dataset used[13]. In our example of the Ras dimer with SOS protein, a residue with a large number of mutations, Q61, was a part of a cluster of spatially close residues, which has other residues with low mutation frequency. The residues with low frequency are within 5Å of the ligand binding site (GDP), highlighting their functional role. However, this method is limited to a good 3D structure, defined experimentally or from homology, and cannot be applied to mutations that occur in intrinsic-disordered regions of the protein, which occur very often in proteins from the Cancer Gene Census.

We have described an approach that will help in predicting those mutations that are damaging and functionally important. This will help in identifying potential driver mutations and prioritize mutations for experimental testing which will ultimately help in guiding drug design.

## Materials and methods

### Protein structure prediction

Where 3D structures are not available for genes, they can be constructed using a variety of software available to search for homologues of known structure and to use appropriate structures as templates for comparative modelling. We have used our in-house modeling pipeline VIVACE[75], which is built in Python using the Ruffus module[76], and combines template searching, single or multiple template alignment, modelling, model quality assessment (NDOPE, GA341, SOAP), optional disordered-region predictions into a single automated program that can be easily parallelized in multiprocessor systems.

In order to identify the homologues, a sequence-structure homology recognition program, FUGUE[77], uses environment-specific substitution tables, which take into account both amino acid sequence information and the local structural environment (secondary structure, solvent accessibility and sidechain interactions) to identify sequences that are compatible with a known protein fold. The search is facilitated by the TOCCATA database (http://mordred.bioc.cam.ac.uk/toccata/), which includes profiles of aligned structures of homologues from the PDB and is organized for use with FUGUE. Originally TOCCATA profiles were for domains assigned from SCOP[78] and CATH superfamilies[79]. The recent VIVACE update now includes all PDBs grouped by CD-HIT[80]. A PSI-BLAST[81] search is run concurrently with FUGUE, thus preventing VIVACE from missing templates that have been submitted to PDB since the most recent CATH and SCOP updates. The total number of PDB domain structures in TOCATTA has increased from 228,000 to 475,000 (the figures refer to the number of structure domains as represented in SCOP[78] and CATH[79]), with many not associated with superfamilies but ensuring access to recent structures. Profiles that share the same CATH–SCOP consensus are also linked together during the template selection phase, where FUGUE is used to consider all the templates in a profile to find the best matches. This is to mitigate the problem of trapping the best template in a mediocre profile.

Following the template-selection phase, up to five of the best templates are picked for alignment using BATON, a streamlined version of the program COMPARER[82]. The resultant alignment is finally used to create the model using MODELLER[83].

An average of ~four models of 202 proteins without crystal structures were produced using the VIVACE pipeline, with ~60% built from more from more than one homologue and with an average FUGUE z-score of 13.97. The average percentage identity of templates, calculated for the final alignment made by BATON between the model and the template structures, was 29.6%, while the average PID of the closest homologue for each gene was 54.2%. Taking only the longest model for each gene, the average coverage is 54.0% and average length 305 residues.

### Mapping mutations on the protein structures

Chimera[84] and PyMol (https://pymol.org/2/) were used to view the 3D-structure of the protein (Table 2) and mutation positions were obtained from the CGC page of the COSMIC database (https://cancer.sanger.ac.uk/census), and the search for the gene name was performed in the search tool. The mutations retrieved from the CGC were then mapped onto the structure.

**Table 2:** Details on the human proteins implicated in cancer, studied here.

Target example	Gene ID (UniProt)	PDB structure	Gene name
Ras Protein and Son of Sevenless hetero complex	P01112 and Q07889	1XD2	HRAS-1
SMAD2	Q15796	1KHX	SMAD2
BRAF and MAP2K1	P15056 and Q02750	4MBJ,4MNE	BRAF and MAP2K1
Androgen receptor	P10275	5CJ6 and 2AM9	AR
Transforming growth factor beta receptor II (TGF-R2)	P15056	1H4I, 3I44, and 1H4J	BRAF
ATP1A1 a sodium/potassium ATPase pump	P05023	2ZXE	ATP1A1

### Predicting effects of mutations

Upon modeling we mapped the mutations from the COSMIC database onto the sequences and 3D-structures to study their effects on protein structure and function using our statistical and machine-learning based methods, namely SDM[31,32] and mCSM[35,36,38] respectively, to measure the effects of mutations on protein stability and protein-protein, protein nucleic acid or protein-ligand interactions.
